# Upregulated Copper Transporters in Hypoxia-Induced Pulmonary Hypertension

**DOI:** 10.1371/journal.pone.0090544

**Published:** 2014-03-10

**Authors:** Adriana M. Zimnicka, Haiyang Tang, Qiang Guo, Frank K. Kuhr, Myung-Jin Oh, Jun Wan, Jiwang Chen, Kimberly A. Smith, Dustin R. Fraidenburg, Moumita S. R. Choudhury, Irena Levitan, Roberto F. Machado, Jack H. Kaplan, Jason X.-J. Yuan

**Affiliations:** 1 Department of Medicine, Section of Pulmonary, Critical Care, Sleep and Allergy Medicine, University of Illinois at Chicago, Chicago, Illinois, United States of America; 2 Department of Pharmacology, University of Illinois at Chicago, Chicago, Illinois, United States of America; 3 Department of Biochemistry and Molecular Genetics, University of Illinois at Chicago, Chicago, Illinois, United States of America; 4 Center for Cardiovascular Research, University of Illinois at Chicago, Chicago, Illinois, United States of America; University of Pittsburgh School of Medicine, United States of America

## Abstract

Pulmonary vascular remodeling and increased arterial wall stiffness are two major causes for the elevated pulmonary vascular resistance and pulmonary arterial pressure in patients and animals with pulmonary hypertension. Cellular copper (Cu) plays an important role in angiogenesis and extracellular matrix remodeling; increased Cu in vascular smooth muscle cells has been demonstrated to be associated with atherosclerosis and hypertension in animal experiments. In this study, we show that the Cu-uptake transporter 1, CTR1, and the Cu-efflux pump, ATP7A, were both upregulated in the lung tissues and pulmonary arteries of mice with hypoxia-induced pulmonary hypertension. Hypoxia also significantly increased expression and activity of lysyl oxidase (LOX), a Cu-dependent enzyme that causes crosslinks of collagen and elastin in the extracellular matrix. In vitro experiments show that exposure to hypoxia or treatment with cobalt (CoCl_2_) also increased protein expression of CTR1, ATP7A, and LOX in pulmonary arterial smooth muscle cells (PASMC). In PASMC exposed to hypoxia or treated with CoCl_2_, we also confirmed that the Cu transport is increased using ^64^Cu uptake assays. Furthermore, hypoxia increased both cell migration and proliferation in a Cu-dependent manner. Downregulation of hypoxia-inducible factor 1α (HIF-1α) with siRNA significantly attenuated hypoxia-mediated upregulation of CTR1 mRNA. In summary, the data from this study indicate that increased Cu transportation due to upregulated CTR1 and ATP7A in pulmonary arteries and PASMC contributes to the development of hypoxia-induced pulmonary hypertension. The increased Cu uptake and elevated ATP7A also facilitate the increase in LOX activity and thus the increase in crosslink of extracellular matrix, and eventually leading to the increase in pulmonary arterial stiffness.

## Introduction

Pulmonary arterial hypertension (PAH) is a severe, progressive disease of the pulmonary circulation manifested by increased pulmonary arterial pressure (PAP) and elevated pulmonary vascular resistance (PVR) that can lead to right ventricular failure and death [1], [2]. The pathogenic mechanism responsible for the elevated of PAP and PVR results from sustained pulmonary vasoconstriction and progressive pulmonary vascular remodeling [3]. The latter involves pulmonary arterial medial hypertrophy due to a combination of excessive proliferation (“muscularization”), decreased apoptosis of smooth muscle cells, adventitial thickening from excessive deposition of collagen and elastin [4], [5], [6], [7], [8], endothelial dysfunction leading to the plexiform arteriopathy [9], and luminal obliteration. Despite our growing knowledge of the pathogenic mechanisms and new targeted therapies for PAH, there are still many questions that remain [10]. A more thorough understanding of PAH development and progression will hopefully lead to novel cellular and molecular mechanisms that can be targeted therapeutically in order to change the course of this life-threatening disease.

Cu is an essential micronutrient; its importance in angiogenesis, wound healing, and anti-oxidant defense has been appreciated for many years. More recently, Cu transporters, ATP7A/B and CTR1, have been identified to be the major membrane proteins that mediate cellular Cu transport. Cu ions have an ability to shift readily between reduced (Cu^+^) and oxidized (Cu^2+^) states, which makes Cu an ideal cofactor in biochemical processes relying on electron transfer, such as respiration (cytochrome c oxidase), anti-oxidation (Cu,Zn-superoxide dismutase (SOD) or SOD1), Fe transport (ceruloplasmin and hephaestin), connective tissue integrity (LOX), among others [11], [12]. At the same time, the propensity of Cu to easily donate and accept electrons makes it potentially toxic through generation of hydroxyl free radicals via Fenton chemistry [13]. For these reasons, it is important that the intracellular Cu concentration is tightly regulated [14].

At the molecular level, Cu has many effects on the pulmonary vasculature that potentially could lead to physiological and pathophysiological variations. Cu,Zn-SOD and eSOD modulate ROS signaling, which has been implicated in vasoreactivity and vascular remodeling [15], [16]. Cu-dependent lysyl oxidase (LOX) is an important contributor to crosslinking of collagen and elastin fibers in the extracellular matrix (ECM) which may promote vascular stiffening/remodeling. LOX is being increasingly recognized as a significant factor contributing to vascular stability and function. Patients with Menkes disease, a disorder where Cu deficiencies (due to mutations in ATP7A) result in inactive LOX, are characterized by a structurally weak, convoluted vasculature leading to aortic aneurysm [17]. LOX is initially synthesized as a 46–48 kDa pro-LOX peptide, and it is then further processed by glycosylation. Incorporation of Cu occurs when passing through the trans-Golgi network. Pro-LOX is secreted from the cell and cleaved by extracellular metalloproteinases, such as BMP-1, into ∼30 kD mature active LOX enzyme [18]. Incorporation of Cu as a cofactor is crucial for activity of the expressed LOX protein, and its activity was shown to be modified in rats by manipulating nutritional Cu levels [18].

Cu enters the cell through the high affinity Cu transporter CTR1 [19], and once inside the cell, it is handled by acceptor-specific Cu-chaperones including Cu chaperone for SOD1 (CCS) that delivers Cu to cytosolic SOD1, COX17 that transfers Cu to mitochondrial cytochrome, and ATOX1 that delivers Cu to ATPases, ATP7A and ATP7B, also known as Menkes and Wilson disease proteins, respectively [20]. Cu-dependent ATPases reside in the Golgi apparatus, transporting Cu inside to be incorporated into newly synthesized proteins (such as pro-LOX, or erythropoietin) bound for secretion [21]. During Cu overload, ATP7A translocates from the Golgi to the plasma membrane to mediate Cu efflux [22], [23]. Recently, White et al. reported that hypoxia is a stimulus for enhanced intracellular Cu transport in murine macrophages [24], while Bogaard et al. described an intriguing link between Cu availability and pulmonary plexiform arteriopathy [25]. Using a rat Sugen5416+hypoxia (SuHx) model of severe PH, the authors were able to prevent the formation of plexiform lesions by restricting dietary Cu content, as well as reverse plexiform lesions and pulmonary hypertension by treating the animals with a Cu chelator, tetrathiomolybdate (TTM) [25]. Hypoxia has also been shown to induce expression of Cu-dependent LOX in metastatic cancer [26]. The goal of this investigation was to examine the role of Cu transport and its downstream effects on LOX in the pathogenesis of chronic hypoxia-induced pulmonary hypertension (HPH).

## Materials and Methods

### Ethics Statement

All animal experiments were conducted according a protocol approved by the University of Illinois at Chicago Institutional Animal Care and Use Committee (Protocol Number 13-086). For hemodynamic measurements, mice were anesthetized with ketamine/xylazine and all efforts were made to minimize suffering.

### Cell culture

Human pulmonary arterial smooth muscle cells (PASMC) obtained from Lonza (Walkersville, MD) were used between passages 7–9. Cells were cultured in M199 medium (CellGro) supplemented with 10% fetal bovine serum (FBS), 20 µg/ml cell growth supplement (BD Biosciences), 25 mg/l of D-valine, 100 IU/ml penicillin and 100 µg/ml streptomycin, in a humidified atmosphere at 37°C and 5% CO_2_. For normobaric hypoxia treatments, cells were incubated for 48–72 hrs in a humidified chamber at 37°C and 5% CO_2_, with 100% N_2_ displacing room air (21% O_2_) down to 3% O_2_, as indicated by an oxygen sensor and controlled by an oxygen pressure control system.

### Chronic hypoxia mouse model of pulmonary hypertension

8-week-old male C57BL/6 mice were exposed to normobaric chronic hypoxia (10% O_2_) or room air (21% O_2_, normoxic control) in a ventilated chamber for 28–35 days according to a protocol approved by the University of Illinois at Chicago Institutional Animal Care and Use Committee (Protocol Number 13-086).

### Hemodynamic measurement and right ventricular hypertrophy

Pulmonary hemodynamic and right ventricular hypertrophy (RVH) measurements were determined as previously described [27]. Briefly, right ventricular pressure (RVP) was measured by a catheter (Millar, Houston, TX) inserted into the right ventricle (RV) via the external right jugular vein. To determine RV hypertrophy, the RV was dissected away from the left ventricle (LV) and septum (S). The Fulton index or the ratio of RV weight to LV+S weight [RV/(LV+S)] was determined and calculated as a measurement for RVH.

### Western blotting

Protein samples were prepared based on the type of protein being analyzed: for cytosol-soluble proteins, cells were lysed in 1×RIPA buffer (BioRad); for integral membrane proteins, cells or tissues were homogenized in HB buffer (150 mM NaCl, 10 mM Tri-HCl saline buffer, pH 7.5, 1 mM EDTA) and spun down at 500 g for 5 min to remove nuclei and unbroken cells, followed by ultracentrifugation at 100,000 g. The supernatant (post-nuclear fraction) was isolated to yield total membranes by ultracentrifugation at 100,000 g. The resulting membrane pellet was resuspended in 1× RIPA buffer. For analysis of HIF-1α, cells or tissues were processed as for total membranes, but the low speed centrifugation step (to remove nuclei) was omitted. Protein concentration was determined and samples were run on SDS gels and transferred to nitrocellulose membranes according to a standard WB protocol. Primary antibodies used: CTR1, ATOX1 (Abcam, ab54865) and LOX (Thermo Scientific, PA 1–16955), HIF-1α (Abcam, ab113642), β-tubulin (Santa Cruz Biotechnology, Inc., sc-9104), and β-actin (Santa Cruz Biotechnology, Inc., sc-81178).

### RT-PCR and Real-time RT-PCR

RNA from cell lysates or tissue homogenates was isolated with TRIzol reagent and quantitated by NanoDrop (Thermo Scientific). 0.5–1 µg of total cellular RNA was taken for reverse transcription with Taq Man Reverse Transcription Reagents (Applied Biosystems), and the resulting first-strand cDNA was used as a template for PCR reaction with High Fidelity Master Mix (New England Biolabs). PCR primers designed to recognize 200–500 bp sequences specific to CTR1, ATP7A, LOX, Cu/Zn-SOD, CCS, and ATOX1 genes were designed at the Invitrogen.com site and subsequently purchased. RT-PCR results were analyzed using 2% agarose gels and ethidium bromide staining. For quantitative Real-Time PCR experiments, SYBER green supermix (Applied Biosciences) was used and results analyzed by the CFX-Manager software (BioRad).

### ^64^Cu uptake

For Cu uptake experiments, PASMC cells were plated in 6-well plates, at 70% confluence. After attaching overnight, cells were divided into two groups, that were either treated, or not, with CoCl_2_ (100 µM CoCl_2_ in M199/10% FBS growth medium) for 48 hrs, or were incubated in a normoxic or hypoxic incubator for 48 hrs prior to the assay. Before initiating the assay, cells were washed twice with PBS and 2 ml of fresh M199 growth medium supplemented with 10% FBS was added to each well and cells were equilibrated with the fresh media for 30 min at 37°C. Cu uptake was initiated by adding 200 µl of 10× [CuCl_2_] labeled with trace amounts of ^64^Cu (MIR radiological sciences, Washington University Medical School). Cells were incubated for the desired times (5 min or 60 min) at 37°C and Cu uptake was terminated by addition of ice-cold stop buffer (150 mM NaCl, 5 mM KCl, 2.5 mM MgCl_2_, 25 mM HEPES, pH 7.4, and 10 mM Na_2_-EDTA), after which cells were washed three additional times with ice-cold stop buffer. Cells were lysed with 1 ml of 1N NaOH and 700 µl of lysates were taken for scintillation counting (Beckman-Coulter LS6500) with Eco-Lume scintillation liquid (ICN Biomedicals #882470). The remaining portion of cell lysates was left until radioactivity decayed, for the determination of total protein concentration in each sample, Bio-Rad Protein Assay (BioRad #500-0006). ^64^Cu transport measurements were carried out in triplicates. ^64^Cu content of the initial tracer-containing buffer was determined for the calculation of specific activity and, following determination of the protein content, Cu uptake was then expressed as picomoles of Cu taken up by the cells per milligram of total cellular protein per minute.

### LOX activity

Activity in the conditioned media and the cell extracts was measured using the Fluorimetric Lysyl Oxidase Assay Kit (AAT Bioquest, Inc., prod. no. 15255). Conditioned media was obtained by culturing PASMC cells for 48–72 hours in phenol red-free M199 medium supplemented with PDGF, under hypoxic or normoxic conditions, and then collecting the growth medium after the incubation period ended. Phenol red-free M199 medium not incubated with the cells was used as a blank. Conditioned media were further concentrated for the LOX assays by centrifugation in commercial protein concentrators (SpinX UF20, Corning, prod. No. 431488). LOX activity was measured according to the manufacturer's instructions, by monitoring LOX-catalyzed H_2_O_2_ release from the fluorescent substrate in an HRP-coupled reaction. To calculate value of Cu-dependent LOX activity, fluorescence of the samples treated with BCS was subtracted from the values of the untreated samples.

### Cell Migration

For a scratch wound assay, PASMC cells were counted and cultured until confluent. A day before the assay, cells were serum starved overnight, washed with PBS, scraped with a sterilized pipet tip, and stimulated with 50 ng/ml of PDGF in M199 medium. Wound closure was monitored for 24 hrs by photographing the same spot (marked with pen) at 0 hr, and then every 6 hrs thereafter, using AM Scope 3.0 camera attached to a light microscope. For the experiment, cells were split into four groups, either treated with or without LOX inhibitor βAPN (Sigma Aldrich, prod. no. M27603), and grown in either a hypoxic or normoxic incubator.

In addition, cell migration was quantitated in Boyden chamber assay, using 24-well Transwells with 8 µm-pores (Corning, Inc., prod. no. 3422). PASMC cell suspensions (5×10^4^ cells), untransfected or transfected with CTR1 siRNA (or scrambled siRNA), were plated on top of the porous membrane, and fresh M199 medium with 0.3% FBS was added to the top and bottom Transwell compartments, supplemented with 200 µM BCS, as indicated. The chamber was incubated at 37°C for 8 hrs. The membrane was fixed and stained using Diff-Quick, and random fields at 200× magnifications were counted.

### Cell proliferation

Bromodeoxyuridine (BrdU) incorporation assay (Calbiochem, prod. no. QIA58) was used to determine cell proliferation. Cells were serum starved overnight, then fresh M199/10% FBS medium was added, and cells were either left untreated (control), or treated with aphidicolin (3 µM), TTM, βAPN, or low serum (0.3%) for the total of 48 hrs. For the last 12 hrs of incubation, BrdU was added to be incorporated into actively dividing cells. Cells were fixed/denatured in ice cold ethanol/0.1N NaOH, and probed with anti-BrdU antibody, followed by HRP-conjugated secondary antibody, and tetramethylbenzidine (TMB) substrate to detect horseradish peroxidase activity. Alternatively, cell proliferation was determined by the degree of expression of proliferating cells nuclear antigen (PCNA), using the Western blot analysis (Santa Cruz, Inc. prod. no. FL-261).

### siRNA treatment

siRNA oligos for HIF-1α, HIF-2α, and CTR1 knockdowns were purchased from Ambion (prod. nos. s6539, s4700, and s3377), and used at final concentration of 100 pmol per 6 well-plate for 48–72 hrs. siRNA was introduced into the cells using RNA Max Lipofectamine reagent (Invitrogen). For the HIF-1α and HIF-2α knockdowns, cells were grown under hypoxic conditions.

### Microaspiration

Micropipette aspiration of attached PASMC was performed as previously described [28]. Briefly, plasma membranes were visualized with a fluorescent membrane dye, carbocyanide DiIC18 (Molecular Probes, Eugene, OR), and then aspirated using micropipettes with 6–9 µm outer diameter pulled from borosilicate glass capillaries (SG10 glass; Richland Glass, Richland, NJ). Zeiss microscopy (Axiovert 200 M) was used for capturing the membrane deformation at multiple time points. Negative pressure was applied to a pipette by a pneumatic transducer tester (BioTek Instruments,Winooski, VT).

### Statistical analysis

Sigma Plot software was used for Student's *t*-test to calculate significance between two groups, and One Way ANOVA for many groups. Composite data are shown as the mean ± S.E. and significant difference is expressed as *P<0.05, **P<0.01, and ***P<0.001.

## Results

### Upregulated mRNA and protein expression of Cu transporters in lung and pulmonary artery tissues isolated from mice with HPH

**Figure 1:**
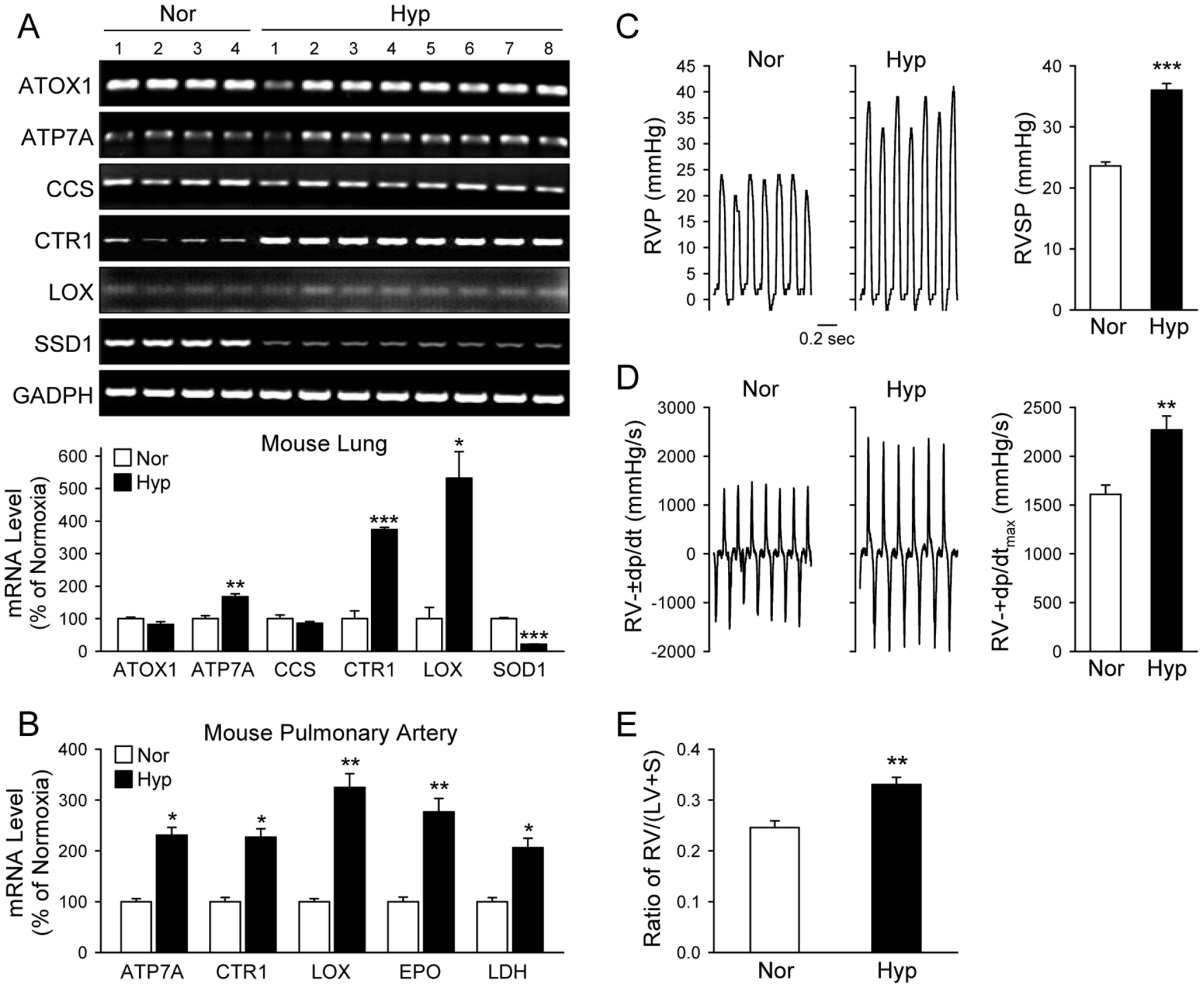
The mRNA expression level of Cu transporters (CTR1 and ATP7A) and lysyl oxidase (LOX) is increased in whole-lung and pulmonary artery (PA) tissues of mice with chronically hypoxia-induced pulmonary hypertension (HPH). Whole lung tissues and isolated PA tissue from normoxic (Nor, 21% O_2_) and hypoxic (Hyp, 10% O_2_ for 5 weeks) mice were homogenized and their mRNA transcripts evaluated by RT-PCR utilizing primers specific for ATOX1, ATP7A, CCS, CTR1, LOX, GAPDH or 18s rRNA (internal controls). A: RT-PCR products from whole-lung tissues were separated on 2% agarose gels (upper panel) and the band intensities quantitated by ImageJ, normalized to intensity of GAPDH, and graphed relative to Nor (n = 4 Nor mouse lungs; n = 8 Hyp mouse lungs). B: PA dissected from Nor and Hyp mice were used for RNA extraction (n = 5) and analyzed by quantitative PCR. Real-time PCR reaction was set with primers specific for the indicated genes. The cycle threshold C(t) values were normalized to 18s rRNA to obtain ΔC(t)_,_ quantified relative to normoxic control for each of the indicated genes (ΔΔC(t)), and graphed as % of normoxic control. C: Representative records of right ventricular pressure (RVP, left panel) and summarized data (mean±SE) showing RV systolic pressure (RVSP) in Nor (n = 6) and Hyp (n = 13) mice. D: Representative records (left panel) and summarized data (right panel, mean±SE) of right ventricular contractility (RV-±dp/dt_max_) in Nor and Hyp mice. E: Summarized data (mean±SE) showing the ratio of right ventricle (RV) weight to left ventricle (LV) and septum (S) weight [RV/(LV+S)] in Nor (n = 7) and Hyp (n = 7) mice. *P<0.05, **P<0.01, ***P<0.001 vs. Nor.

To examine whether Cu transportation is involved in the development of HPH, we first measured and compared mRNA and protein levels of the Cu transporters (CTR1 and ATP7A), Cu chaperones (ATOX1 and CCS) and Cu-dependent enzymes (LOX) in lung tissues from normoxic and hypoxic mice. As shown in Figure 1, the mRNA expression level of CTR1, ATP7A and LOX in lung tissues was significantly upregulated in chronically hypoxic mice (10% O_2_, for 4 weeks) in comparison to control mice (21% O_2_, for 4 weeks). The mRNA levels of the Cu chaperones, ATOX1 and CCS, however, remained unchanged in lung tissues isolated from mice chronically exposed to hypoxia (Fig. 1A upper and lower panels). The real-time RT-PCR experiments indicated that the mRNA expression levels of CTR1, ATP7A and LOX in isolated pulmonary arteries were all significantly higher in hypoxic (Hyp) mice than in normoxic (Nor) mice (Fig. 1B). Lactase dehydrogenase (Ldh) and erythropoietin (Epo) were used as control genes known to be upregulated by hypoxia (Fig. 1B) [29].

Chronically hypoxic mice exhibited significantly higher right ventricular systolic pressure (RVSP, 36.01±1.09 vs. 23.59±0.66 mmHg; *P*<0.001, Fig. 1C), greater right ventricular contractility (RV-dp/dt_max_, 1609.46±305.52 vs. 2268.78±322.54 mmHg/s; *P*<0.01, Fig. 1D) and greater Fulton index (0.330±0.014 vs. 0.246±0.013; *P*<0.01, Fig. 1E) than normoxic controls. These data indicate that the upregulated mRNA expression of CTR1, ATP7A and LOX in whole-lung tissues and isolated pulmonary arteries is associated with HPH.

Western blot analysis of whole lung tissues was then used to determine whether increased mRNA expression of CTR1 and LOX resulted in a corresponding increase in CTR1 and LOX protein expression level. CTR1 is an integral membrane protein with a relatively low basal expression. To determine the protein expression level of CTR1 in the plasma membrane, we concentrated lung tissue homogenates by spinning down total membranes and then solubilizing membrane proteins for the Western blot experiments. Using a previously characterized c-terminal CTR1 antibody [30] and a commercially available LOX antibody (Sigma), we recognized a glycosylated and non-cleaved form of LOX (pro-LOX ∼58 kD) and a processed LOX (∼35 kD).

**Figure 2:**
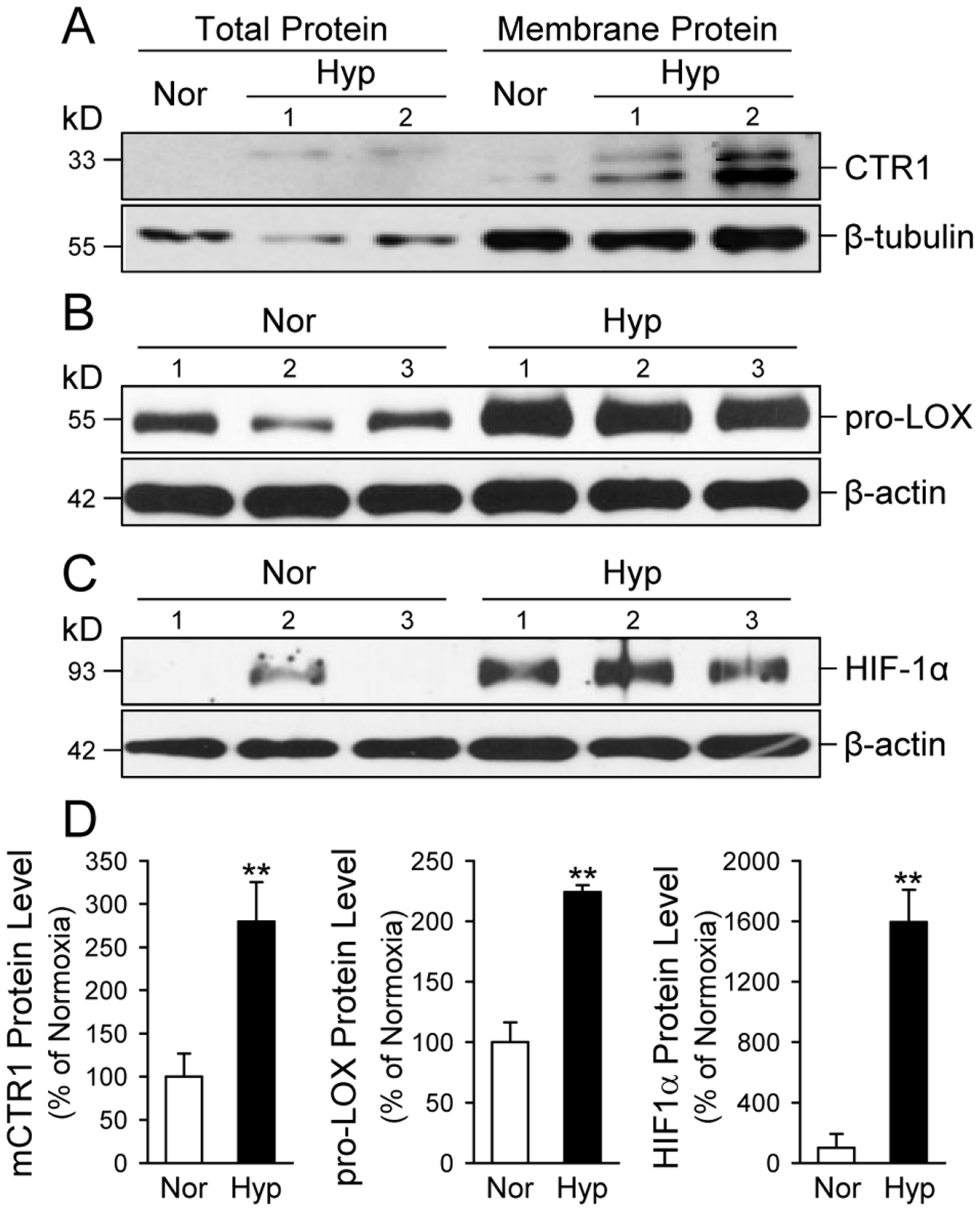
The protein expression level of CTR1, pro-LOX and HIF-1α is increased in whole-lung tissues of mice with HPH. A–C: Western blot analysis of mouse CTR1 (A), pro-LOX (B) and HIF-1α (C) in total and membrane proteins extracted from whole-lung lung tissues of normoxic control mice (Nor, n = 5) and chronically hypoxic (Nor, n = 5) mice. Proteins from Nor and Hyp mouse lungs were solubilized in 3% DDM/1× RIPA buffer and utilized for Western blot analysis using antibodies specific for mouse CTR1, pro-LOX, and HIF-1α. β-actin or β-tubulin was used as a loading control. D: Summarized data (mean±SE) showing protein expression levels of CTR1, pro-LOX and HIF-1α in lungs tissues isolated from Nor and Hyp mice. The band intensity was quantitated with ImageJ software, normalized with respect to the loading control, and then shown relative to control (% of Nor). **P<0.01 vs. Nor.

As shown in Figure 2, the protein expression levels of CTR1 and pro-LOX in whole lung tissues from mice with HPH were significantly higher than in lung tissues from normoxic control mice (Fig. 2A, 2B and 2D, left panels). The upregulated expression of CTR1 and pro-LOX was associated with a significantly upregulated expression of HIF-1α (Fig. 2C and 2D, right panel). The protein level of HIF-1α in lung tissues of hypoxic animals was approximately 14 times the level in lung tissues of normoxic control animals (Fig. 2C and 2D, right panel), while the intensity of CTR1 band (at 33 kD) in hypoxic mice was approximately 2.2 times higher than in normoxic mice (Fig. 2D, left panel). Lysyl oxidase (LOX) was detected mostly as a 58-kD pro-LOX band which was approximately 2.1 fold higher in hypoxic mouse lungs than in normoxic control lungs (Fig. 2D, middle panel). These results indicate that both mRNA and protein expression levels of CTR1 and pro-LOX were significantly increased in lung tissues of mice with HPH.

### Hypoxia and CoCl_2_ upregulate mRNA expression of Cu transporters and increases Cu uptake in human PASMC

**Figure 3:**
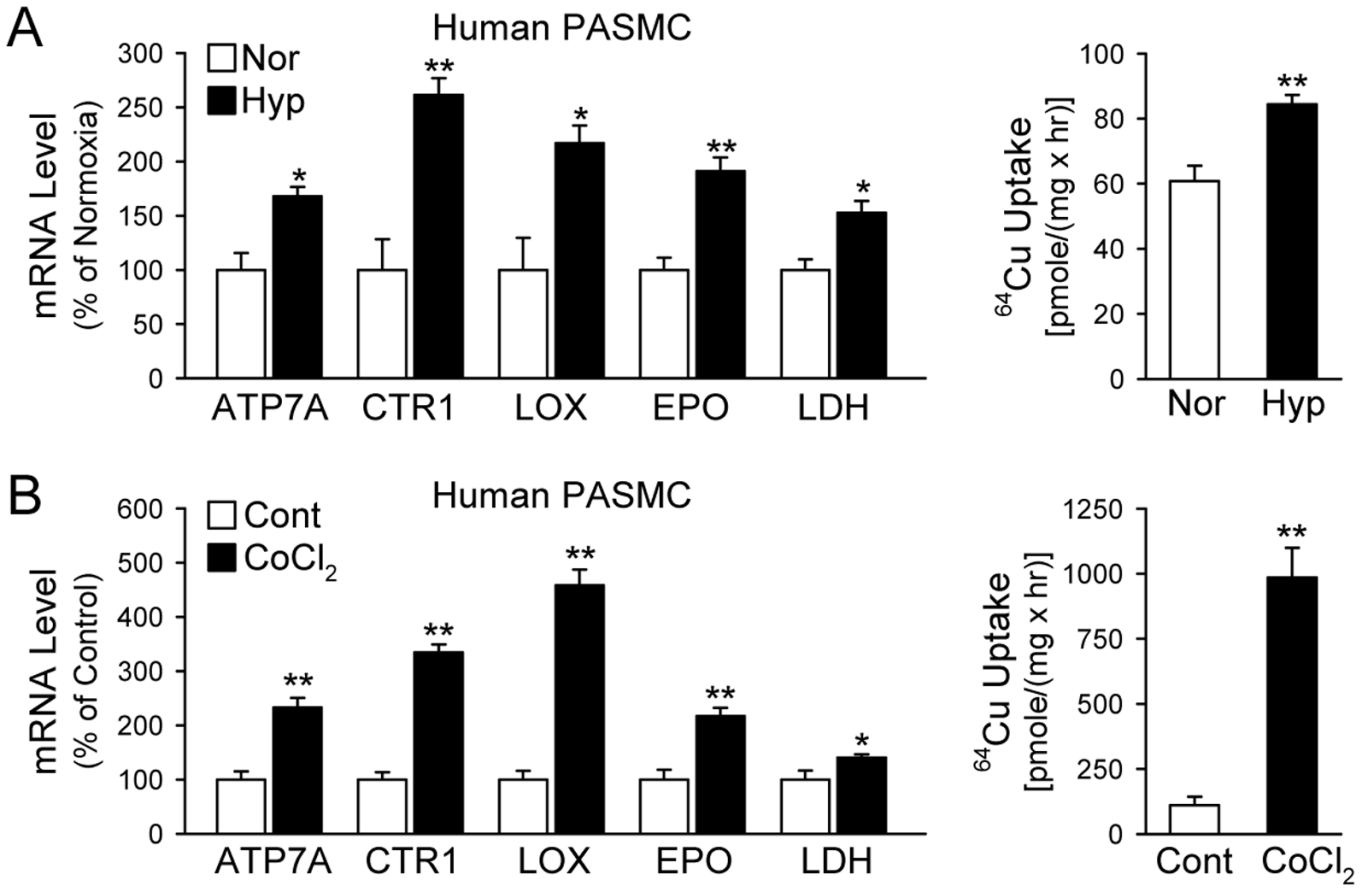
Hypoxia-mediated upregulation of mRNA expression of Cu transporters (CTR1, ATP7A) and lysyl oxidase (LOX) is associated with an increase in Cu transportation in human pulmonary arterial smooth muscle cells (PASMC). A: Real-time RT-PCR analysis on ATP7A, CTR1, and LOX (left panel) and ^64^Cu uptake (mean±SE) in human PASMC exposed to normoxia (Nor) and hypoxia (Hyp, 3% O_2_ for 48 hrs, n = 3; right panel). B: Real-time RT-PCR analysis on ATP7A, CTR1, and LOX (left panel) and ^64^Cu uptake (mean±SE, right panel) in human PASMC treated with vehicle (Cont) and CoCl_2_ (100 µM for 48 hrs, n = 3; right pane). Lactate dehydrogenase (LDH) and erythropoietin (EPO) were used as positive controls. *P<0.05, **P<0.01, ***P<0.001 vs. Hyp or CoCl_2_.

In addition to the in vivo experiments shown above, we conducted in vitro experiments to examine whether hypoxic exposure of human PASMC exerts the same effect on the Cu transporters and chaperones. Our real-time RT-PCR experiments showed that incubation of PASMC under hypoxic conditions (3% O_2_ for 48 hrs), or treatment of PASMC with CoCl_2_, which can also increase HIF-1α level to activate the HIF-1α-dependent signaling cascades [31], significantly increased the mRNA expression level of ATP7A, CTR1 and LOX in PASMC (Fig. 3A and 3B, left panels). We used lactase dehydrogenase (Ldh) and erythropoietin (Epo) as control genes known to be upregulated by hypoxia (Fig. 3, left panels) [29].

To determine whether the hypoxia-induced increase in CTR1 expression in PASMC results in an increase in transportation of Cu, we measured and compared ^64^Cu uptake in PASMC under normoxic and hypoxic conditions. Incubation of PASMC under hypoxic condition (3% O_2_ for 48 hrs) significantly enhanced ^64^Cu uptake by approximately 30% (Fig. 3A, right panel). Treatment of PASMC with 100-µM CoCl_2_ (for 48 hrs) led to a dramatic increase in the uptake of ^64^Cu (by approximately 16 fold) (Fig. 3B, right panel). These observations indicate that the upregulated expression of CTR1 was associated with a significantly increased Cu uptake in PASMC. The hypoxia- (or CoCl_2_-) induced increase in CTR1 expression and Cu uptake results in a significant increase in the amount of Cu in PASMC.

### HIF-1α is involved in hypoxia-mediated upregulation of CTR1 mRNA expression in PASMC

**Figure 4:**
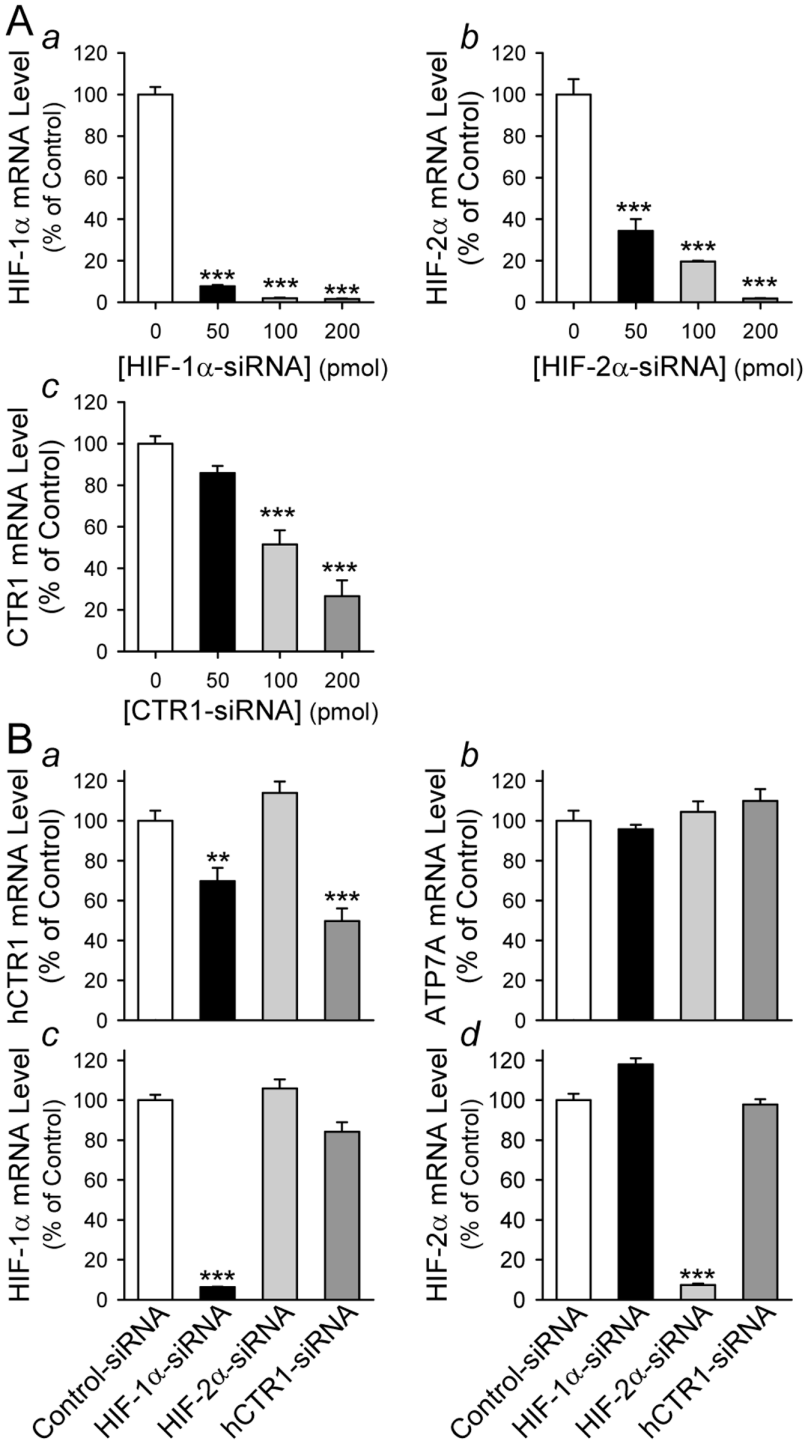
Downregulation of HIF-1α by siRNA significantly attenuates mRNA expression of CTR1 in hypoxic PASMC. A: Real-time RT-PCR analysis on HIF-1α (*a*), HIF-2α (*b*) and CTR1 (*c*) in hypoxic PASMC treated with (50–200 pmol) or without (0 pmol) siRNA specifically targeting HIF-1α, HIF-2α and CTR1, respectively. Data are shown in mean±SE. ***P<0.01 vs. control hypoxic cells (0-pmol siRNA). B: Real-time RT-PCR analysis on human CTR1 (*a*), ATP7A (*b*), HIF-1α (*c*) and HIF-2α (*d*) in hypoxic PASMC treated with 100-pmol scrambled siRNA (Control-siRNA, open bars), HIF-1α-siRNA (solid bars), HIF-2α-siRNA (light grey bars), and CTR1-siRNA (dark grey bars), respectively. ***P<0.01 vs. hypoxic cells treated with scrambled siRNA (Control-siRNA).

Recently, HIF-2α was found to be important for transcription of the Cu-ATPase, ATP7A [32]. To examine whether HIF-1α or HIF-2α is necessary for hypoxia-mediated upregulation of CTR1 and ATP7A, we compared mRNA expression levels of CTR1 and ATP7A in PASMC during hypoxia (and in PASMC treated with CoCl_2_) [31]. To optimize the experimental condition, we first conducted a dose-response experiment for two specific siRNA we used to knockdown HIF-1α and HIF-2α. As shown in Figure 4A, the siRNA (at the doses of 50, 100 and 200 pmol) for HIF-1α (Hif-1α-siRNA) efficiently decreased mRNA level of Hif-1α (Fig. 4A *a*), while the siRNA (at the doses of 50, 100 and 200 pmol) for HIF-2α (Hif-2α-siRNA) significantly decreased Hif-2α mRNA level (Fig. 4A *b*) in PASMC incubated under hypoxic condition. The inhibitory effect of the siRNA for CTR1 (CTR1-siRNA) on CTR1 mRNA level was dose-dependent; 50 pmol of CTR1-siRNA seemed to be insufficient to knockdown CTR1, while 100 and 200 pmol of CTR1-siRNA significantly decreased CTR1 mRNA level (Fig. 4A *c*) in PASMC. Interestingly, we found that downregulation of HIF-1α with Hif-1α-siRNA only significantly decreased mRNA level of CTR1 in hypoxic PASMC, but not ATP7A mRNA level (Fig. 4B *a*-*d*). Knockdown of HIF-2α with Hif-2α-siRNA, however, had no effect on either CTR1 or ATP7A (Fig. 4B *a*–*d*). These results indicate that HIF-1α is potentially a transcription factor involved in hypoxia-mediated upregulation of CTR1 in PASMC, whereas hypoxia-mediated upregulation of ATP7A may be due to a HIF-1α/2α-independent mechanism.

### Hypoxia increases lysyl oxidase activity

As described above, hypoxia upregulates CTR1, enhances Cu uptake and leads to an increase in the amount of Cu in PASMC. A fraction of the increased cellular Cu would be delivered to ATP7A at the Golgi membrane and then incorporated into newly synthesized pro-LOX protein, which traffics through the trans-Golgi network to be secreted out of the cell. To examine whether upregulated CTR1 and augmented Cu uptake lead to increased LOX secretion, we measured the activity of LOX protein secreted from PASMC under hypoxic conditions by collecting and concentrating the conditioned culture medium. Values for each sample were normalized to the total protein concentration in the conditioned medium, and an irreversible LOX inhibitor, β-aminopropionitrile (βAPN), was used as negative control. The amount of Cu-dependent LOX activity was calculated by subtracting LOX activity of the samples in the presence of a Cu chelator, bathocuproine disulphonate (BCS), from the total LOX activity.

**Figure 5:**
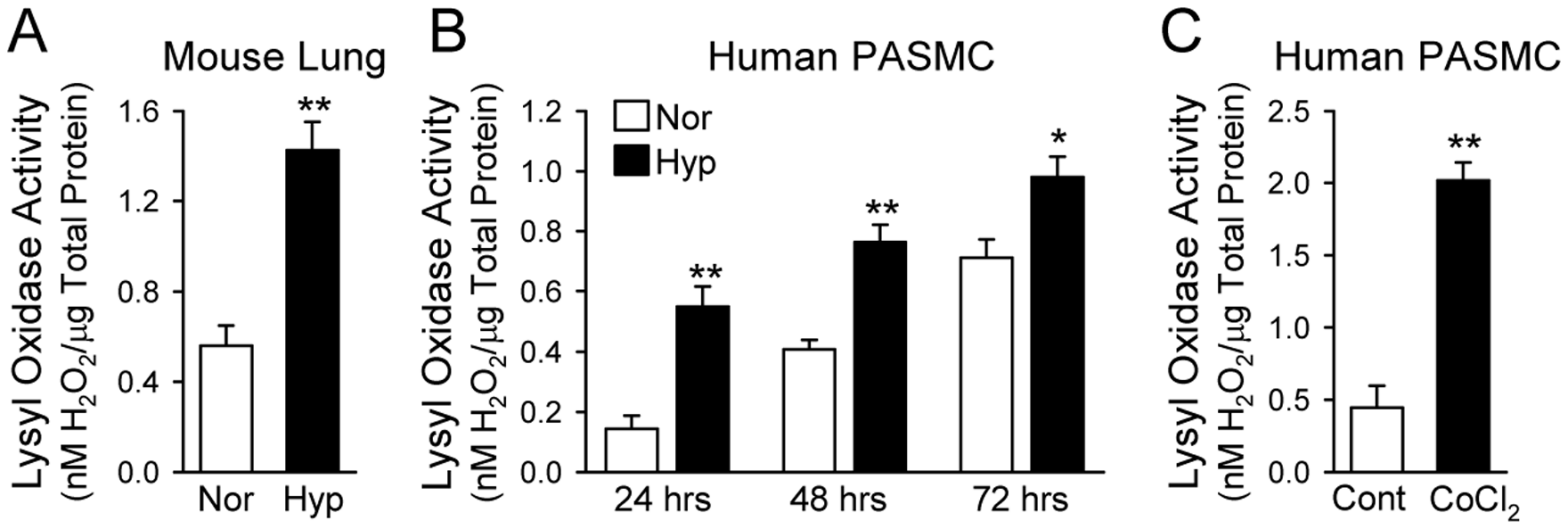
Activity of lysyl oxidase (LOX) is increased in whole-lung tissues of chronically hypoxic mice and in culture media from human PASMC exposed to hypoxia or treated with CoCl_2_. Enzymatic activity of LOX was determined using Fluorimetric Lysyl Oxidase Assay Kit by monitoring LOX-catalyzed H_2_O_2_ release from the fluorescent substrate in HRP-coupled reaction. A: LOX activity in lung tissue homogenates from normoxic (Nor, room air for 5 weeks, n = 5) and hypoxic (Hyp, 10% O_2_ for 5 weeks, n = 5) mice. **P<0.01 vs. Nor. B: LOX activity in culture media collected from human PASMC after 24, 48 and 72 hrs of exposure to normoxia (Nor, 21% O_2_) or hypoxia (Hyp, 3% O_2_). *P<0.05, **P<0.01 vs. Nor. C: LOX activity in culture media collected from PASMC treated with vehicle (Cont) or CoCl_2_ (100 µM for 48 hrs). **P<0.01 vs. Cont. Each bar graph displays the Cu-dependent activity of LOX determined by subtracting values obtained in the presence of BCS (a Cu chelator) from values in the absence of BCS. LOX activity is expressed in nanomoles of H_2_O_2_ released from the cells and normalized to the amount of total protein in each sample. Data are shown as mean±SE.

As shown in Figure 5A, there was an approximately 2.3 fold increase in LOX activity in supernatants of lung tissue homogenates isolated from hypoxic mice in comparison to normoxic control mice. Similarly, the in vitro experiments also indicated that enzymatic activity of LOX in conditioned media collected from PASMC incubated under hypoxic condition (Fig. 5B) or treated with CoCl_2_ (Fig. 5C) was significantly higher than in normoxic PASMC and cells treated with vehicle.

### Hypoxia significantly increases Cu-dependent PASMC migration

**Figure 6:**
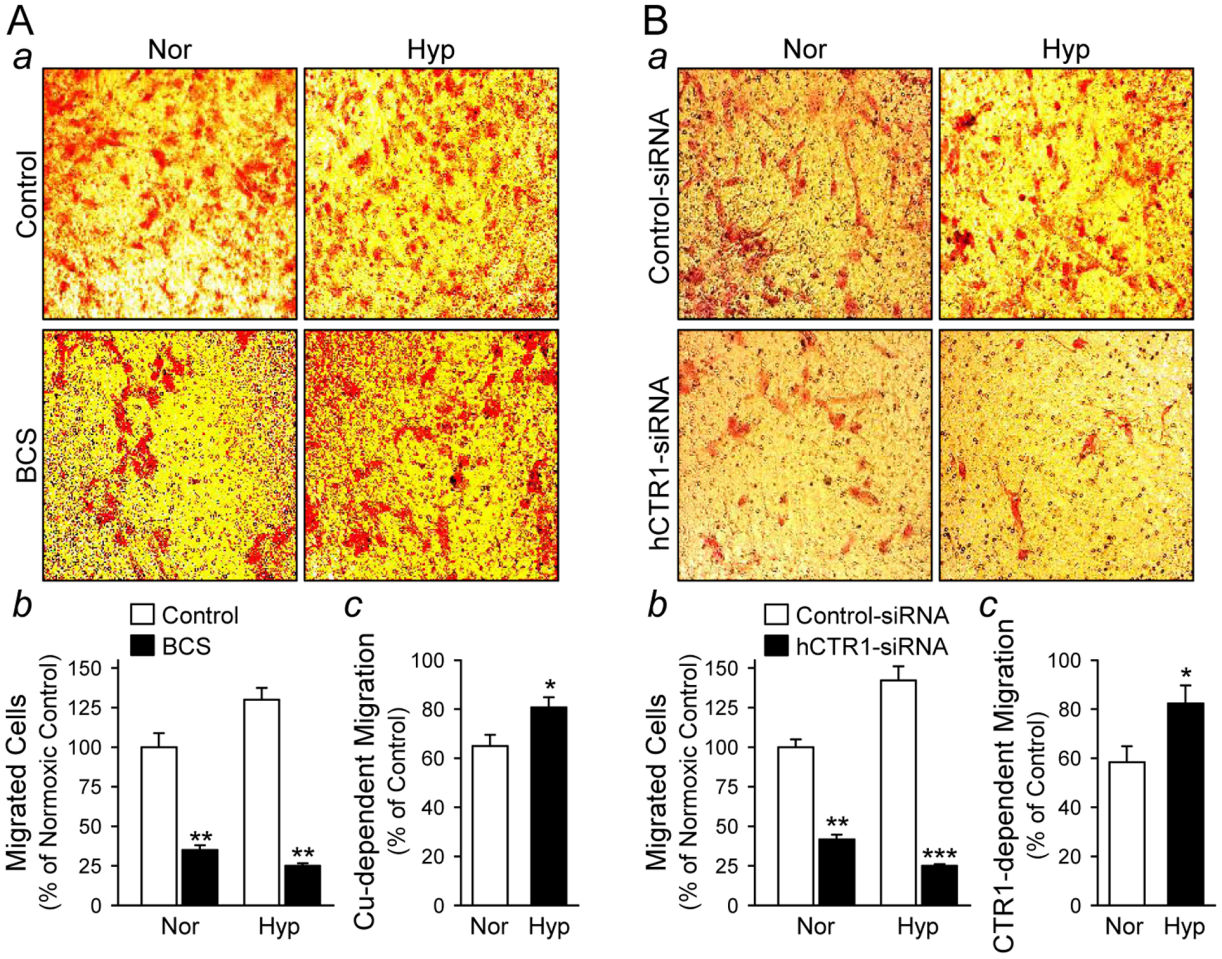
PASMC migration is dependent on Cu and inhibited by knockdown of CTR1. Cell migration was determined by the modified Boyden chamber assay. Cells were plated on top of the porous (8-µm pore) membrane. After 48 hrs, the membrane was fixed and stained using Diff-Quick and the migrated cells in randomly chosen fields were counted at 200× magnification. A: Representative images (*a*) showing human PASMC cultured under normoxic (Nor) or hypoxic (Hyp) conditions in the absence (Control) or presence (BCS) of 200 µM BCS (a Cu chelator). Summarized data (mean±SE) showing migrated cell counts (*b*) in normoxic (Nor) and hypoxic (Hyp) PASMC treated with (BCS) or without (Control) BCS. **P<0.01 vs. Control. The Cu-dependent cell migration (*c*) was determined by the percent changes in migrated cell counts between Control and BCS-treated cells in Nor and Hyp. Data are shown as mean±SE. *P<0.05 vs. Nor. B: Representative images (*a*) showing human PASMC treated with scrambled siRNA (Control-siRNA) or CTR1-siRNA under Nor or Hyp conditions. Summarized data (mean±SE) showing migrated cell counts (*b*) in Nor and Hyp PASMC treated with Control-siRNA (solid bars) or CTR1-siRNA (open bars). **P<0.01 vs. Control. The Cu-dependent cell migration (*c*) was determined by the percent changes in migrated cell counts between PASMC treated with Control-siRNA and CTR1-siRNA in normoxia (Nor) and hypoxia (Hyp). Data are shown as mean±SE. *P<0.05 vs. Nor.

The next set of experiments was designed to define the role of Cu in PASMC migration. Boyden chamber migration and scratch wound assays were utilized to examine whether CTR1-dependent Cu affects PASMC migration incubated under hypoxic condition. Typical smooth muscle cell culture medium (e.g., M199) contains approximately 1 µM Cu [33]. To examine the effect of this basal amount of Cu on cell migration, we treated cells with or without a Cu chelator, BCS, under normoxic and hypoxic conditions. In the Boyden chamber migration assay, Cu chelation dramatically inhibited PASMC migration under both normoxic (Nor) and hypoxic (Hyp) conditions (Fig. 6A *a* and *b*); Cu-dependent migration (Control minus BCS treatment) was significantly greater in PASMC exposed to hypoxia in comparison to PASMC exposed to normoxia (Fig. 6A *c*, *P*<0.05). Consistent with the inhibitory effect of Cu chelation by BCS, knockdown of CTR1 with siRNA led to a comparable decrease in cell migration (Fig. 6B *a* and B*b*), which was again significantly greater in hypoxic compared to normoxic PASMC (Fig. 6B *c*, *P*<0.05). These data indicate that hypoxia-mediated increase in the Cu uptake via CTR1 contributes to enhanced PASMC migration.

**Figure 7:**
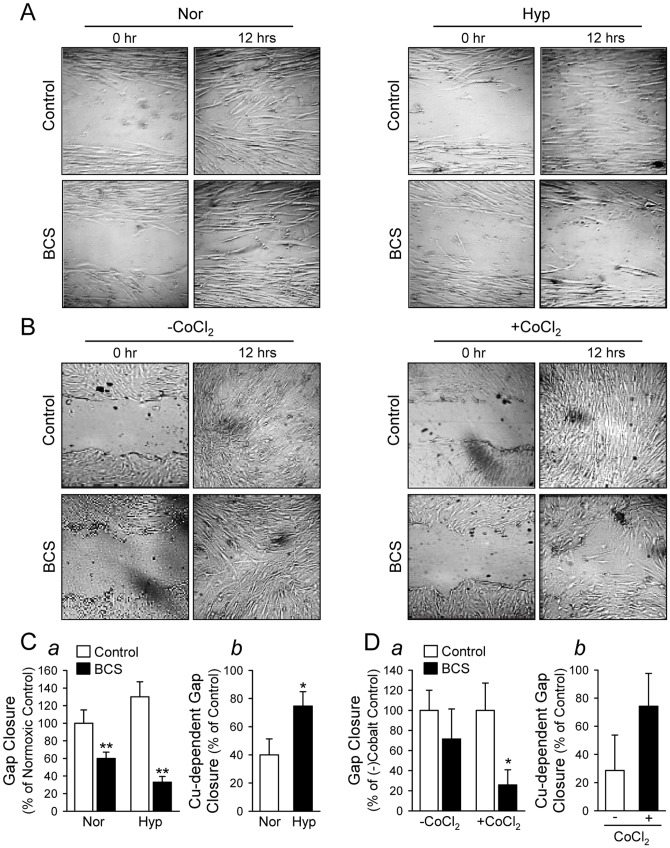
PASMC motility is dependent on Cu and the Cu-dependent PASMC motility is augmented by hypoxia. Cell motility was determined by a scratch wound assay over a period of 12: Representative images showing normoxic (Nor) and hypoxic (Hyp) PASMC immediately (0 hr) or 12 hrs after scratch with a sterile pipette in the absence (Control) or presence (BCS) of 200 µM BCS (a Cu chelator). B: Representative images showing control (-CoCl_2_) and 100-µm CoCl_2_-treated PASMC immediately (0 hr) or 12 hrs after scratch in the absence (Control) or presence (BCS) of BCS. C: Summarized data (mean±SE) showing gap closure (*a*) measured at 12 hr in Nor and Hyp PASMC treated with (BCS) or without (Control) BCS. **P<0.01 vs. Control. The Cu-dependent cell motility (*b*) was determined by the percent changes in gap closure between Control and BCS-treated PASMC under Nor and Hyp conditions. *P<0.05 vs. Nor. D: Summarized data (mean±SE) showing gap closure (*a*) measured at 12 hr in PASMC treated with (+CoCl_2_) or without (-CoCl_2_) CoCl_2_ in the absence (Control) or presence (BCS) of BCS. *P<0.05 vs. Control. The Cu-dependent cell motility (*b*) was determined by the percent changes in gap closure between Control and BCS-treated PASMC in the absence (-) or presence (+) of CoCl_2_.

In addition, our results obtained for the scratch wound assay also show a significant decrease in PASMC motility when cells were treated with BCS (Fig. 7). Chelation of Cu with BCS significantly decreased PASMC motility (determined by gap closure) in both normoxic and hypoxic PASMC (Fig. 7A and 7C *a*); an effect that was more pronounced under hypoxic conditions (Fig. 7C *b*). PASMC motility was also assessed with and without CoCl_2_ treatment and a significant decrease in motility was observed with Cu chelation after treatment with CoCl_2_ (Fig. 7B and 7D *a*). There was a trend toward decreased migration without the addition of CoCl_2_ and though CoCl_2_ treatment does appear to show greater Cu-dependent migration, no statistical differences were observed due to a wide variability in outcomes (Fig. 7D *b*).

### Cu chelation attenuates proliferation of PASMC and decreases expression of the anti-apoptotic protein Bcl-2

**Figure 8:**
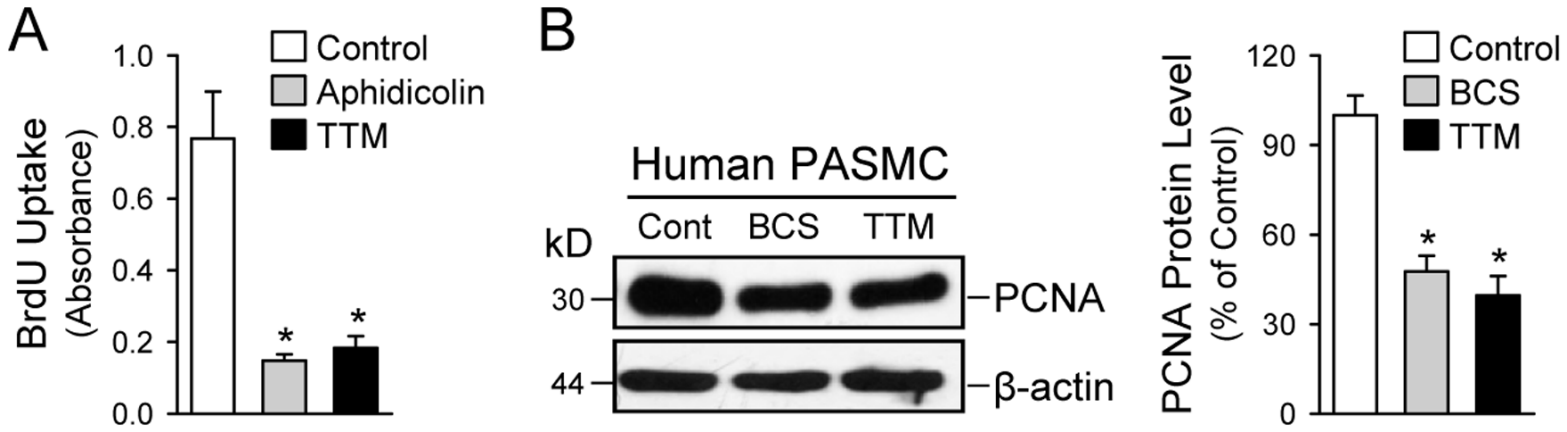
Chelation of Cu diminishes human PASMC proliferation. A: BrdU incorporation (mean±SE) in control PASMC and PASMC treated with aphidcolin (a DNA-replication inhibitor, 48 hrs) and 100 µM tetrathiomolybadate (TTM, a Cu chelator, 48 hrs). *P<0.05 vs. Control. Cells treated with aphidicolin were used as a negative control. B: Western blot analysis (left panel) on PCNA (a proliferating cell nuclear antigen) in control (Cont) PASMC and PASMC treated with the Cu chelators, BCS and TTM. Summarized data (right panel, mean±SE) showing PCNA protein levels in control cells, BCS-treated cells and TTM-treated cells. *P<0.05 vs. Control.

Next, the effect of Cu and CTR1 on the proliferation of PASMC cells was evaluated by the bromodeoxyuridine (BrdU) incorporation assay, as well as by the Western blot analysis with anti-proliferating cell nuclear antigen (PCNA) antibody. Figure 8A indicates that the treatment of PASMC with 100 µM tetrathiomolybdate (TTM), a cell permeable Cu chelator, results in a dramatic decrease of BrdU incorporation, comparable to the aphidicolin treatment (an inhibitor of DNA replication, used as the negative control here). Similar to the case of BrdU assay, the expression of PCNA was decreased in cells treated with either BCS or TTM, indicating that Cu is required for cell proliferation (Fig. 8B, left and right panels). We anticipated that TTM would have a greater inhibitory effect on proliferation than BCS because TTM can cross the plasma membrane [34], and should chelate the intra- and extra-cellular Cu and BCS is a membrane-impermeable compound [34], and therefore should only bind the extracellular Cu. Both compounds (BCS and TTM) had a similar inhibitory effect on the PCNA expression (Fig. 8B). It is likely that TTM does not further inhibit proliferation because it is not able to out-compete the Cu-chaperones and glutathione for Cu binding [35], as all of the intracellular Cu is either protein- or glutathione-bound [36], [37].

**Figure 9:**
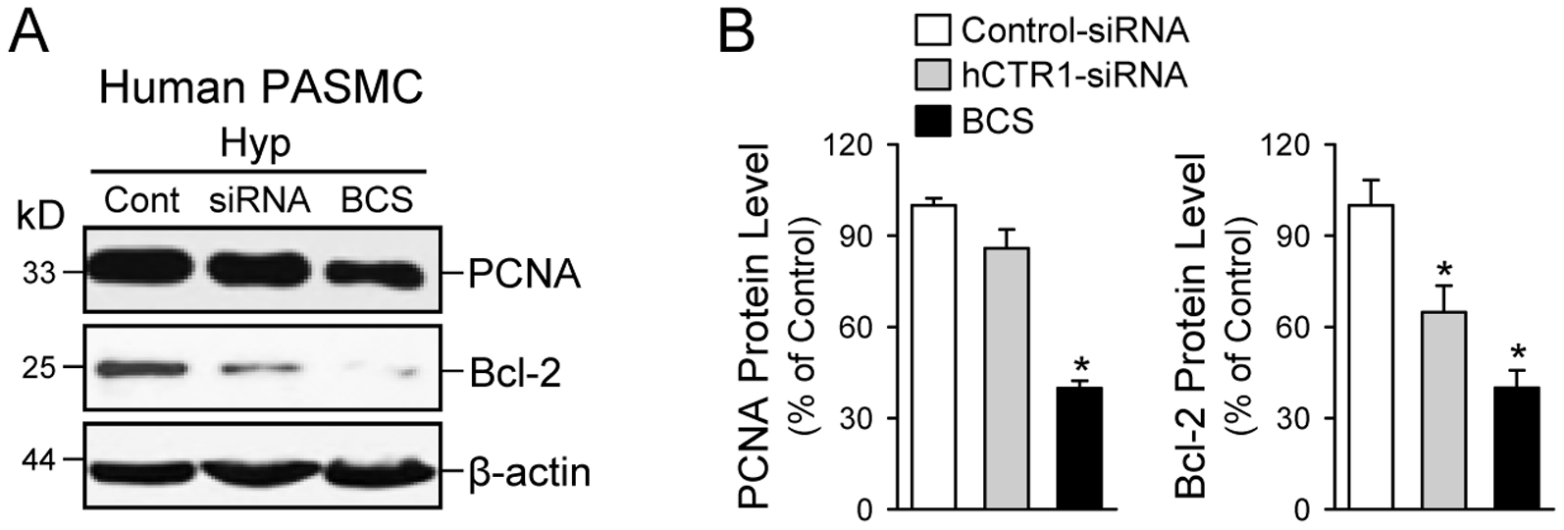
Chelation of Cu and knockdown of CTR1 both decrease Bcl-2 expression in human PASMC during hypoxia. A: Western blot analysis on PCNA (a marker for cell proliferation) and Bcl-2 (an anti-apoptotic protein) in PASMC transfected with scrambled siRNA (Cont) or human CTR1-siRNA (siRNA) and PASMC treated with the Cu chelator BCS. B: Summarized data (mean±SE) showing PCNA (left panel) and Bcl-2 (right panel) protein levels in PASMC transfected with Control-siRNA or hCTR1-siRNA and PASMC treated with BCS. *P<0.05 vs. Control-siRNA.

In addition to the level of PCNA, we examined the effect of BCS on the expression of Bcl-2, an anti-apoptotic protein, during hypoxia (Fig. 9A). We found that Cu-chelation (by BCS), not only downregulated protein expression level of PCNA (Fig. 9A), but also significantly decreased levels of Bcl-2 protein by ∼60% (Fig. 9B). These data indicate that Cu ions, or inward transportation of Cu ions, are important for activation of cell survival or anti-apoptotic signals (determined by Bcl-2 expression level) and for cellular proliferation (determined by PCNA expression level).

### Knockdown of CTR1 exerts apoptotic effect on PASMC

To investigate whether inward transportation of Cu ions through CTR1 is involved in PASMC proliferation and survival, we also measured and compared the expression levels of PCNA (a marker for cell proliferation) and Bcl–2 (an anti-apoptotic protein) in human PASMC treated with control siRNA (Cont) and siRNA specifically targeting human CTR1 (hCTR1-siRNA). The real-time RT-PCR experiments showed that 100 µM of hCTR1-siRNA treatment decreased CTR1 mRNA level by 45–55% in PASMC (data not shown). As shown in Figure 9, knockdown of CTR1 with siRNA (hCTR1-siRNA) in PASMC during hypoxia negligibly affected the protein expression level of PCNA, but significantly decreased the protein expression level of Bcl–2 (by approximately 36%, *P*<0.05 vs. cells treated with control siRNA). Chelation of Cu with BCS, a membrane impermeable Cu chelator, significantly decreased the protein expression level of both PCNA and Bcl–2 in PASMC during hypoxia (Fig. 9). These data indicate that, while Cu ions are required for or involved in exerting pro-proliferative (determined by PCNA expression level) and anti-apoptotic (determined by Bcl-2 expression level) effects on PASMC, transportation of Cu ions through CTR1 seems to be involved only in upregulation of Bcl-2 in PASMC (or the anti-apoptotic effect on PASMC) based on the downregulating effect of BCS on Bcl-2 as shown in Figure 9. The results further imply that CTR1 plays a predominant role in cell survival (potentially via its upregulating effect on the anti-apoptotic protein Bcl-2 since downregulation of CTR1 with siRNA only decreased Bcl–2 level but negligibly affected PCNA level, as shown in Figure 9B) while its role in cell proliferation can be compensated, possibly by an alternative mechanism of cellular Cu uptake.

### Inhibition of lysyl oxidase inhibits proliferation and enhances apoptosis in PASMC

**Figure 10:**
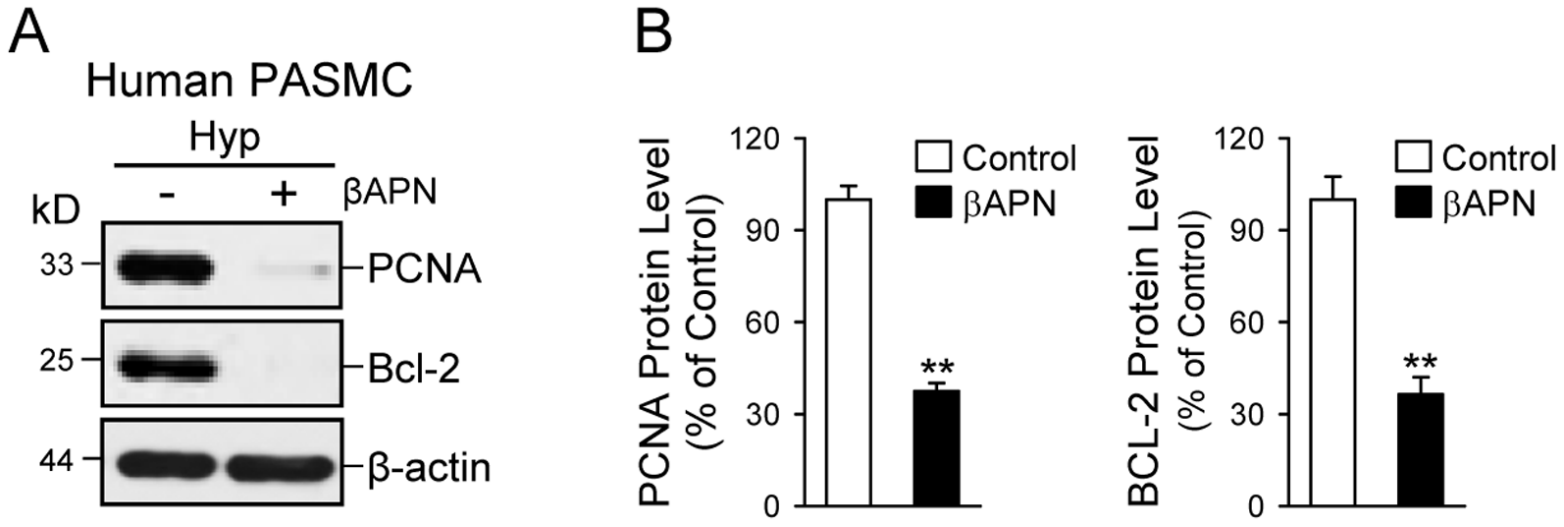
Inhibition of LOX downregulates PCNA (a marker for cell proliferation) and Bcl–2 (an anti-apoptotic protein) in human PASMC during hypoxia. A: Western blot analysis on PCNA and Bcl–2 in hypoxic PASMC treated with (+) or without (-) the irreversible LOX inhibitor βAPN (for 48 hrs). B: Summarized data (mean±SE) showing PCNA (left panel) and Bcl–2 (right panel) protein levels in control PASMC (open bars) and PASMC treated with βAPN (solid bars). **P<0.01 vs. Control.

Lysyl oxidase (LOX) regulates extracellular matrix organization by crosslinking collagen and elastin, and increased activity (and/or secretion) of LOX increases ECM rigidity or stiffness [38]. The next set of experiments was designed to examine whether blockade of LOX affects PASMC proliferation and migration. As shown in Figure 10, treatment of human PASMC during hypoxia with β-aminopropionitrile (βAPN, for 48–72 hrs) significantly decreased the protein expression level of PCNA (a marker for cell proliferation) and Bcl–2 (an anti-apoptotic protein). These results imply that LOX is involved in regulating PASMC proliferation and survival by affecting extracellular matrix and cellular stiffness.

### IPAH-PASMC have increased pro-LOX and show increased cellular stiffness

As shown above, hypoxia-induced pulmonary vascular remodeling is, at least in part, associated with *a*) HIF-1α-dependent upregulation of CTR1 and pro-LOX in PASMC, and *b*) LOX-mediated extracellular matrix and cellular stiffness. To confirm that LOX-mediated cellular stiffness is not only a mechanism partially involved in the development of hypoxia-mediated pulmonary hypertension, but also a potential mechanism for idiopathic pulmonary arterial hypertension (IPAH), we measured and compared deformability or stiffness of PASMC isolated from normal subjects and IPAH patients using micropipette aspiration or microaspiration.

**Figure 11:**
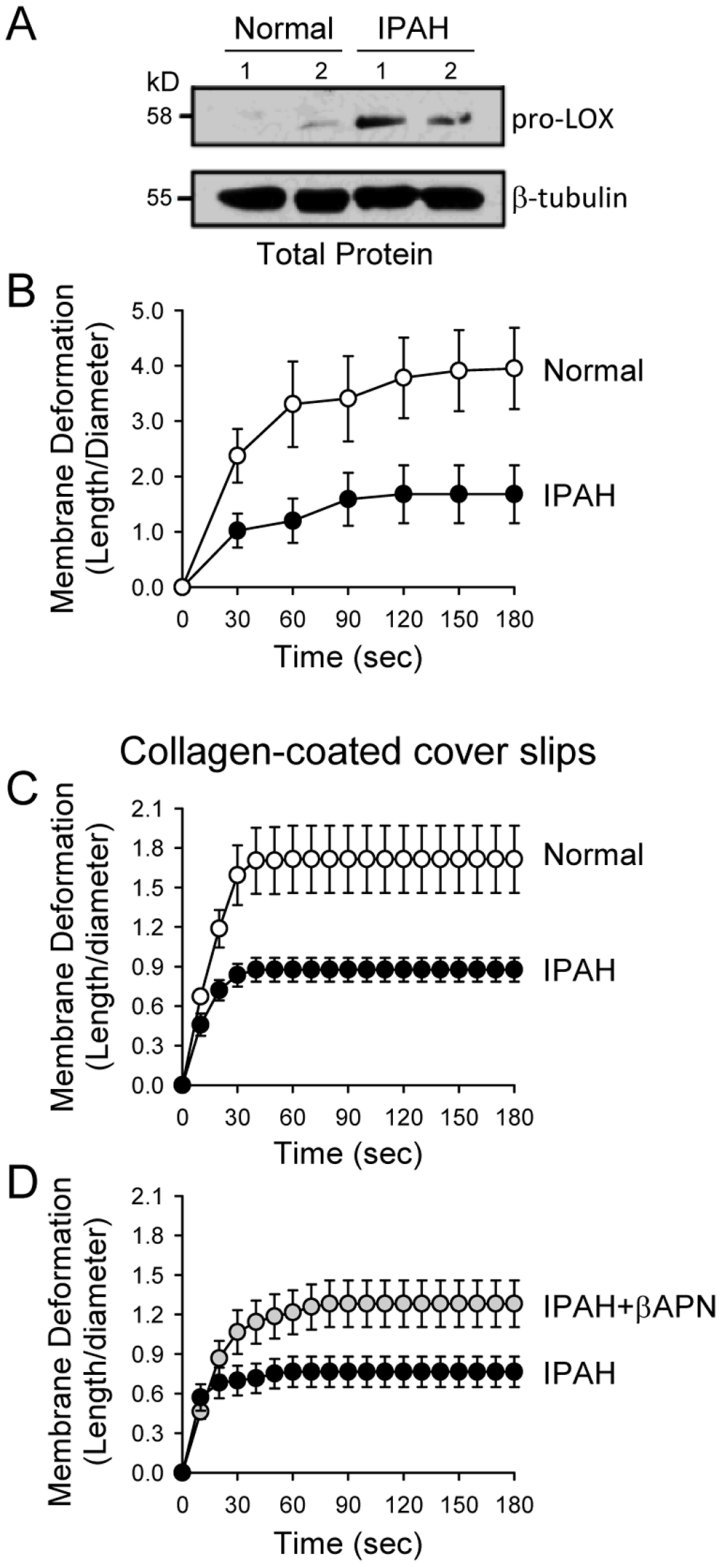
Upregulated pro-LOX expression in PASMC isolated from patients with idiopathic pulmonary arterial hypertension (IPAH) is associated with increased stiffness. A: Western blot analysis on pro-LOX in PASMC from normal subjects (Normal) and IPAH patients (IPAH). B: Time courses of the membrane deformation in normal PASMC (open circles) and IPAH-PASMC (closed circles) cultured on uncoated cover slips. Microaspiration was used to determine the membrane deformation which is inversely related to membrane stiffness. *P*<0.01 between the two curves (using two-way ANOVA). C: Time courses of the membrane deformation in normal PASMC and IPAH-PASMC cultured on collagen-coated cover slips. *P*<0.01 between the two curves (using two-way ANOVA). D: Time courses of the membrane deformation in IPAH-PASMC treated with (IPAH+βAPN) or without (IPAH) the LOX inhibitor βAPN. *P*<0.01 between the two curves (using two-way ANOVA).

Microaspiration is a simple method to estimate cell stiffness by measuring the degree of membrane deformation in response to negative pressure applied by a glass micropipette to the cell surface. Using microaspiration, investigators have shown previously that plasma membrane composition, such as the cholesterol content [39], the level of oxidized oxLDL [40], [41], and sub-membrane cytoskeleton [28], [42] all affect the degree of cellular stiffness and membrane deformation [43]. Here, we show that PASMC derived from IPAH patients (IPAH-PASMC) exhibited significantly higher expression of pro-LOX than normal PASMC (Fig. 11A). Deformation of cells measured by microaspiration was significantly decreased in IPAH-PASMC compared to normal PASMC, implying a greater overall stiffness of IPAH-PASMC cultured on glass cover slips (Fig. 11B) or collagen-coated cover slips (Fig. 11C). Inhibition of LOX activity with β-aminopropionitrate (βAPN) significantly increased the degree of membrane deformation (i.e., significantly decreased the stiffness) of IPAH-PASMC cultured on collagen-coated cover slips, implying that LOX may contribute to the increased cell stiffness in IPAH-PASMC (Fig 11D).

## Discussion

Pulmonary vascular remodeling and increased stiffness of pulmonary arteries both contribute to the development and progression of pulmonary hypertension. In this study, we identified a novel mechanism in which chronic hypoxia caused increased Cu transport and LOX activity that promoted the development of pulmonary hypertension in a murine model. In lung tissues and isolated pulmonary arteries from animals with HPH and in PASMC exposed to hypoxia, our data indicated that mRNA and protein expression level of the high affinity Cu transporter CTR1 was significantly upregulated in comparison to normoxic controls. The hypoxia-mediated upregulation of CTR1 seemed to be mediated by a HIF-1α-dependent mechanism because downregulation of HIF-1α significantly attenuated the hypoxia-mediated CTR1 upregulation. The upregulated CTR1 was functionally associated with an increased Cu uptake in PASMC exposed to hypoxia, and the increased intracellular Cu contributed to augmenting PASMC proliferation, survival and migration during hypoxia.

Our data also showed that hypoxia upregulated the Cu export pump ATP7A and the Cu-dependent enzyme LOX in lung tissue homogenates, isolated pulmonary arteries and cultured PASMC. The hypoxia- (or CoCl_2_-) mediated increase in expression of ATP7A was, however, due to a HIF-1α-independent mechanism because downregulation of HIF-1α had negligible effect on hypoxia-mediated ATP7A upregulation. Upregulated LOX expression and increased Cu-LOX activity usually lead to crosslink of collagen and elastin in extracellular matrix and increase in pulmonary arterial stiffness (or PASMC stiffness) which, in combination with the increased PASMC proliferation and migration, further contributes to the development and progression of HPH. By identifying upregulation of the entire Cu transport cascade (CTR1, ATOX1, ATP7A, and LOX), the data from this study demonstrate that Cu and Cu-dependent enzymes may play an important role in HPH and the Cu transporters may be good targets to develop therapeutic approaches for pulmonary hypertension.

Since the identification and cloning of CTR1 [44], various studies have characterized the kinetics of CTR1-mediated Cu transport and the structural properties of CTR1, such as topology, mapping of glycosylation sites, identifying amino acids essential for transport, and determining the 3D structure using cryoelectron microscopy [19], [30], [45], [46]. Despite this knowledge, information is still very limited regarding the cellular regulation of Cu homeostasis and transport in mammalian cells. Recently, White et al. reported a novel regulatory mechanism of Cu homeostasis in a mammalian macrophage cell line (RAW 264.7) that was dependent on hypoxia [47].

Similar to macrophages, PASMC are uniquely equipped to sense O_2_ tension and orchestration of cellular responses to hypoxia is mediated in mammals by two isoforms of hypoxia-inducible factors, HIF-1α and HIF-2α. HIF-1α mediates primarily an acute response to hypoxia, while HIF-2α is most often thought to require a chronic stimulus to accumulate, and becomes more important during the late phases of hypoxia [48]. In the present study, we were able to knockdown both HIF-1α and HIF-2α with respective siRNA, but only HIF-1α-siRNA attenuated CTR1 mRNA levels in PASMC exposed to 3% O_2_ for 48 hrs. Examination of the promoter region of the CTR1 gene found the presence of the putative hypoxia-responsive element (HRE) (5′-cgtg-3′), a recognition binding site for HIF-1α/2α [49]. More extensive studies are needed to investigate whether and how HIF-1α transcriptionally regulates the CTR1 gene in PASMC.

It has recently been shown that excess Cu stabilizes HIF-1α [50], [51]. Together with the data mentioned above that HIF-1α is involved in hypoxia-mediated upregulation of CTR1 and, subsequently, increase in intracellular Cu, we speculate that a positive feedback loop or chain reaction exists in hypoxic PASMC. Hypoxia activates HIF-1α and upregulates CTR1 expression via HIF-1α, and then increases intracellular Cu which may subsequently stabilize HIF-1α and further potentiate the HIF-1α-mediated upregulation of CTR1. This scenario may not occur physiologically, as the intracellular Cu concentration capable of exerting a positive effect on HIF-1α activation is 50–125 µM, which is much higher than the normal physiological intracellular Cu concentration (less than 5 µM). During hypoxia (or when HIF-1-α is activated and CTR1 is upregulated), intracellular Cu concentration may rise to the level that can exert the positive effect on HIF-1α leading to sustained activation of HIF-1α and maintain a prolonged effect of hypoxia on PASMC proliferation and migration. A previous study demonstrated that that Cu depletion using a Cu-chelation decreased the binding of HIF-1α to its downstream HRE domain-containing target genes, by inhibiting the formation of complexes between HIF-1α and DNA [51].

In rats with severe pulmonary hypertension (induced by Sugen and hypoxia), Bogaard et al. [25] recently showed that Cu chelation with TTM or Cu-restricted diet caused regression of established pulmonary vascular intimal lesions and prevented formation of new intimal lesions through inhibition of pulmonary arterial endothelial cell (PAEC) proliferation. Their histological examination, however, showed that Cu restriction had no effect on pulmonary vascular thickening attributable to the medial hypertrophy (due mainly to excessive PASMC proliferation). These observations suggest that Cu is mainly involved in stimulating PAEC proliferation causing intimal lesions, but has little effect on stimulating PASMC proliferation. By determining BrdU incorporation and PCNA expression, we showed in our in vitro study that Cu chelation led to a dramatic inhibition of PASMC proliferation, while knockdown of CTR1 with siRNA also resulted in attenuation of PASMC proliferation (and migration) during hypoxia. Furthermore, inhibition of LOX has been proposed to treat hypertensive and fibrotic disorders [52], [53] and metastatic cancers [54]. In PASMC cultured in hypoxia, inhibition of LOX activity with βAPN also significantly attenuated proliferation indicating that increased Cu-dependent LOX activity is involved in stimulating PASMC proliferation.

In summary, the data from this study imply that increased Cu transport due to upregulated CTR1 (and ATP7A) contribute to the increase in PASMC proliferation and migration, which subsequently cause pulmonary vascular remodeling characterized by medial and adventitial hypertrophy. The enhanced Cu uptake and elevated ATP7A expression in PASMC may also contribute to increasing pulmonary arterial stiffness by potentiating Cu-LOX activity and collagen/elastin crosslink. Development of drugs targeting the Cu-CTR1-LOX signaling axis may be a novel strategy to develop more efficient therapeutic approaches for pulmonary hypertension associated with hypoxia.
